# The Impact of *ACE* Gene Variants on Acute-Phase Reactants in Children with Rheumatic Heart Disease

**DOI:** 10.3390/diagnostics13101672

**Published:** 2023-05-09

**Authors:** Abdulhadi H. Almazroea, Sondos Yousef, Salma M. S. Ahmad, Hanin N. AlHiraky, Amal Al-Haidose, Atiyeh M. Abdallah

**Affiliations:** 1 Pediatrics Department, College of Medicine, Taibah University, Al-Madinah 30001, Saudi Arabia; amazreoa@taibahu.edu.sa; 2 Department of Biomedical Sciences, College of Health Sciences, QU Health, Qatar University, Doha 2713, Qatar; sy1701619@student.qu.edu.qa (S.Y.); sa1701249@student.qu.edu.qa (S.M.S.A.); ha1601407@student.qu.edu.qa (H.N.A.);

**Keywords:** *ACE*, angiotensin-converting enzyme, polymorphism, C-reactive protein, erythrocyte sedimentation rate, rheumatic fever, rheumatic heart disease

## Abstract

Rheumatic heart disease (RHD) is the most important sequela of upper respiratory group A *Streptococcus* (GAS) infection. The role of the common angiotensin-converting enzyme (*ACE*) insertion/deletion (I/D) variant in the disease and its subtypes remains uncertain. The acute-phase reactants (APRs) C-reactive protein (CRP) and erythrocyte sedimentation rate (ESR) form part of the Jones criteria for diagnosing RHD, and genetic factors are known to influence baseline CRP and ESR levels. Therefore, here, we investigated the relationship between the *ACE* I/D polymorphism and APR levels in RHD. A total of 268 individuals were recruited, including 123 RHD patients and 198 healthy controls. There was a trend toward a higher D allele frequency in RHD patients. The *ACE* I/D polymorphism genotype frequency and DD+ID allelic carriage were significantly associated with a high APR level (*p* = 0.04 and *p* = 0.02, respectively). These results highlight the importance of *ACE* I/D polymorphisms in RHD for disease stratification, but not for disease predisposition. Further studies in larger cohorts and different populations are now required to confirm this association and to explore the mechanism of this effect.

## 1. Introduction

Rheumatic heart disease (RHD) usually occurs during childhood after group A streptococcal infection. In response to the infection, an uncontrolled immune response in genetically susceptible hosts causes rheumatic fever (RF) and inflammation that severely damages the heart valves, thus causing RHD [1]. RHD is often associated with poverty and poor healthcare services, so it disproportionately affects low- and middle-income countries (LMICs) whilst having been almost completely eradicated from high-income countries [2]. The burden of RHD increased from 15 million cases in 2005 to 40.5 million cases in 2019 [3]. Although penicillin is used to treat group A streptococcal infections and as a preventative medicine, the disease still affects many young people in LMICs [4].

The genetic predisposition to RHD is well documented [5]. Genome-wide association and case–control studies have identified many important genes significantly associated with RHD, many of which have immunological functions such as immunoglobulin heavy chain alleles (*IGHV4-61*), *IL10*, and macrophage migration inhibitory factor (*MIF*) (for recent reviews, see [5,6]). Moreover, there is a reported association between RHD subtypes and clinical laboratory findings. Some genes have been reported to influence disease severity based on the subgrouping of patients according to valve involvement, e.g., MVLs (mitral valve lesions) and CVLs (combined valve lesions). For example, in a cohort of patients from Saudi Arabia, *MIF* polymorphisms were associated with the MVL but not CVL subtype of the disease [7].

Clinical heterogeneity is a well-known feature of RF and RHD. Patients experience the first symptoms at different ages, and different organs can be involved. There is no specific diagnostic test for RF and RHD, and the diagnosis is based on clinical criteria and the exclusion of alternative diagnoses. The Jones criteria, first introduced in 1944, are the most commonly used clinical criteria for the diagnosis of RF and carditis [8]. Investigation for major and minor Jones criteria involves echocardiography and laboratory testing, and, in most cases, a diagnosis of recent streptococcal infection is required. Carditis, joint complaints, subcutaneous lumps, and chorea are among the major criteria [9], while clinical signs such as fever and erythema marginatum, stomach pain, epistaxis, and pulmonary abnormalities and laboratory indicators of acute inflammation such as leukocytosis and a high erythrocyte sedimentation rate (ESR) or high C-reactive protein (CRP) make up the minor criteria [10].

Acute-phase reactants (APRs) describe a group of changes, metabolic and systemic, appearing as a rapid response to inflammatory stimuli. These reactants or responses reflect protective and adaptative mechanisms occurring before the immune response [11]. Most APRs are proteins (~30) upregulated in response to abnormalities including myocardial infarction, bacterial infection, trauma, and collagen tissue disorders. These conditions lead to the production of pro-inflammatory cytokines (IL-1, IL-6, TNF-α). APRs remain circulating at high concentrations if the inflammatory response is not self-limiting, and the greater the inflammatory response, the higher the APR levels [12].

The most common APRs and those most used in clinical practice are CRP and ESR [13,14]. In inflammatory states, CRP can increase many thousandfold, especially in severe cases [15]. Not all acute-phase proteins increase in all inflammatory reactions. For instance, in systemic lupus erythematosus, CRP decreases but ESR increases. Furthermore, CRP responds quicker to the inflammatory reaction, while ESR has a slower response and does not tend to increase over the first 24–48 h of an inflammatory reaction [16]. Other APRs are not commonly quantified because their levels tend to be highly heterogeneous (even in the same population), they are difficult to measure, and the changes can persist [12]. Nevertheless, CRP and ESR levels are known to be influenced by the underlying genetics in different diseases [17,18]. For example, in a recent GWAS, several variants in different diseases were associated with baseline ESR levels [19]. Moreover, complement receptor 1 gene variants influenced baseline ESR levels in patients treated with anti-TNF therapy in a UK rheumatoid arthritis cohort [20,21].

Angiotensin-converting enzyme (ACE), a zinc-dependent peptidase, is an important and necessary component of the renin–angiotensin system (RAS) responsible for the conversion of angiotensin I (Ang I) to the vasoconstrictor Ang II. *ACE* is located on human chromosome 17q23 and is involved in many physiological processes such as blood pressure control, hematopoiesis, reproduction, renal development, and immune responses. ACE inactivation can suppress tumor growth and angiogenesis and decrease cardiovascular mortality [22,23]. Genetic variability in *ACE* has been shown to influence ACE protein levels in different populations. *ACE* has 26 exons and 25 introns, and the insertion/deletion (I/D) polymorphism in intron 16 is the most investigated variant, characterized by the I/D of an Alu repeat sequence that is 287 non-coding base pairs long. This *ACE* variant is associated with ACE protein expression variability in the peripheral blood. Carriers of the D/D genotype have the highest plasma levels of ACE, while those with genotype I/I have the lowest. Heterozygous individuals (I/D genotype) have moderate plasma protein levels of ACE, suggesting combined control. Previously, we reported a weak association between the *ACE* I/D polymorphism and RHD [24]; however, other studies did not detect this association [25,26]. Moreover, while there have been studies of the association between *ACE* polymorphisms and APR levels in different diseases such as pulmonary and heart diseases, there has yet to be such a study in RHD. We hypothesized that changes in ACE activities under the influence of ACE polymorphisms may alter baseline APR levels. Therefore, the main aim of this study was to investigate the association between *ACE* polymorphisms and APR levels in RHD patients.

## 2. Materials and Methods

### 2.1. Patients and Laboratory Testing

This was a case–control study of 123 unrelated Saudi Arabian RHD patients recruited from the Pediatric Cardiology Clinic at the Maternity and Children Hospital, Medina, Saudi Arabia. The patient and control groups were described previously [7,24,27]. Laboratory measurements, including baseline ESR and CRP, were performed on all patients as part of the clinical assessment according to the Maternity and Children Hospital laboratory standard operating procedure. RF was diagnosed according to the modified Jones criteria at initial diagnosis [8] and confirmed via echocardiography. Elevated APR was defined as patients with an ESR > 22 mm/h for men and 27 mm/h for women and elevated CRP > 20 mg/L. Exclusion criteria were patients with other heart complications, inflammatory conditions, and RF without valve involvement.

The Centre for Genetics and Inherited Diseases (CGID) research ethics committee and the Maternity and Children Hospital ethics committee approved the study, which followed the World Medical Association Declaration of Helsinki. All adult patients and donors and the parents/guardians of participants <18 years old signed a fully informed and written consent approved by the committees. The control group comprised 198 age-, gender-, and ethnicity-matched unrelated healthy blood donor volunteers without evidence or a family history of cardiac diseases or any autoimmune diseases.

### 2.2. Sample Preparation and Genotyping

DNA samples were extracted from 2 mL peripheral blood using the QIAamp DNA Mini Kit (Qiagen, Hilden, Germany) according to the manufacturer’s protocol. Extracted DNA was quantified via spectrophotometry (MaestroNano, MaestroGen, Las Vegas, NV, USA). DNA samples were stored at −80 °C until use. The genotyping method was previously reported [28]. Briefly, primers flanking the insertion/deletion region (forward primer 5′-CTG GAG ACC ACT CCC ATC CTT TCT-3′ and reverse primer 5′-GAT GTG GCC ATC ACA TTC GTC AGA T-3′) were used to detect the presence or absence of the 287 bp sequence in intron 16 of *ACE* [28]. Amplification was carried out in a 12.5 μL reaction volume containing 30 ng genomic DNA added to 2× Go-Taq master mix including MgCl_2_, 10× PCR buffer, dNTPs, and 10 units of Taq DNA polymerase (Cat #M7132, Promega, Madison, WI, USA). A Veriti thermal cycler was used to amplify the PCR product (Life Technologies, Carlsbad, CA, USA). PCR cycles were as follows: initial denaturation at 95 °C for 2 min, followed by 35 cycles with denaturation at 95 °C for 30 s, annealing at 58 °C for 15 s, and extension at 72 °C for 30 s. PCR products were visualized using 2% agarose gels and ethidium bromide staining under UV light (G:BOX, Syngene, Bangalore, India). Samples with an insertion allele (I) produced a 490 bp PCR fragment; a deletion allele (D) produced a 190 bp product without the 287 bp Alu insertion. In heterozygous samples, two PCR fragments (490 and 190 bp) were detected. Randomly selected samples were repeated to confirm the accuracy of the method. Genotypes were determined by direct counting and labeled DD homozygous when only the 190 bp fragment was present, II homozygous when only the 490 bp fragment was present, and ID heterozygous when both fragments were present.

### 2.3. Statistical Analysis

The genotype and allele frequencies were determined by direct counting in patients and controls. Differences in genotype distributions of the polymorphism between cases and controls were analyzed using chi-squared contingency table analysis or Fisher’s exact test, as appropriate. Odds ratios (OR) and 95% confidence intervals (CIs) were calculated. A *p*-value < 0.05 was considered statistically significant. All genotyping data were checked for any deviation from the Hardy–Weinberg equilibrium via chi-squared testing. Statistical analysis of the data was performed using SPSS v.24 (IBM, Chicago, IL, USA).

## 3. Results

### 3.1. Patient Characteristics

The clinical records of 123 RHD patients attending the Pediatric Cardiology Clinic at the Maternity and Children Hospital, Medina, Saudi Arabia, were reviewed. Seventy-five patients had an elevated baseline APR (ESR > 22 mm/h for men, 27 mm/h for women; CRP > 20 mg/L). The clinical characteristics of all patients and the control group are shown in Table 1. There was no significant difference in age and gender between the control and patient groups.

### 3.2. Association with RHD

Table 2 shows the distribution of *ACE* I/D polymorphism genotypes, allele frequencies, and allelic carriages in the RHD patients and control group. All frequencies were in Hardy–Weinberg equilibrium. The genotype distribution of *ACE* I/D was not significantly different between patients and controls. However, the DD+ID allelic carriage was higher in patients than controls, with patients having a 2.9-fold increased risk of RHD than those with the II genotype (*p* = 0.04, OR = 2.9, 95% CI: 1.1–7.6).

### 3.3. Association with ARP

**Figure 1:**
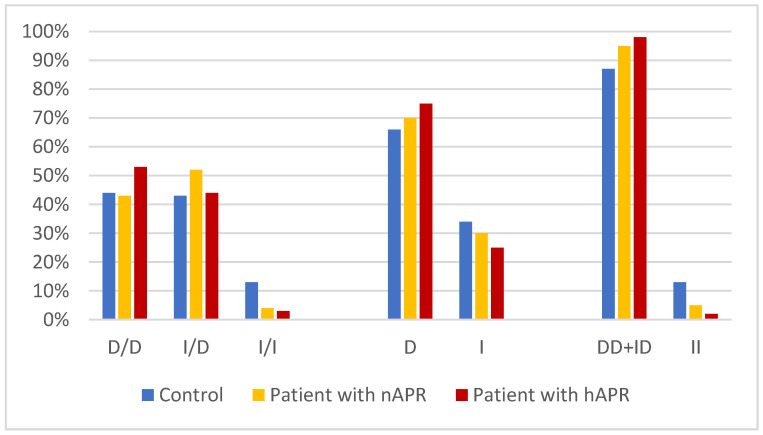
Bar chart showing the distribution of the *ACE I/D* polymorphism frequencies in the RHD and control groups. A significant distribution of the ACE I/D genotype frequency, D allele frequency, and DD+ID allele frequency between patients with high APR levels and controls was found (*p* = 0.04, *p* = 0.04, and *p* = 0.02, respectively). RHD, rheumatic heart disease; APR, Acute-phase reactants; nAPR, normal APR; hAPR, high APR; ACE, Angiotensin-converting enzyme; D, deletion allele; I, insertion allele.

The RHD group was divided into two subgroups according to APR levels (high and normal), which were compared with the control cohort. The genotypes, allele frequencies, and allele carriage distributions of these three groups are shown in Table 3. There was a significant difference in the distribution of the *ACE* I/D genotype frequency, D allele frequency, and DD+ID allele frequency between patients with high APR levels and controls (*p* = 0.04, *p* = 0.04, and *p* = 0.02, respectively) but not between patients with normal APR levels and controls. There were no differences in allele frequency between patients with high and normal APR levels. Figure 1 presents a bar chart showing the distribution of the *ACE* I/D polymorphism frequencies in the RHD and control groups.

### 3.4. Association with Disease Subtypes

To examine whether the ACE D allele was associated with disease severity, we investigated the association between the *ACE* I/D allele and valvular heart involvement. Table 4 shows the distribution of *ACE* I/D genotypes in patient subgroups and controls. Carrying the *ACE* D allele was significantly associated with MVLs compared with controls (*p* = 0.02). However, there was no significant difference in the CVL subgroup.

## 4. Discussion

The rationale for studying the *ACE* I/D polymorphism is based on strong evidence that it plays a role in modulating immune responses and modifies clinical outcomes in other diseases [29]. Previously, we reported a weak association between RHD and the *ACE* I/D polymorphism [24]. However, in this study, we investigated the association between the *ACE* I/D polymorphism and baseline APR levels in RHD patients. RHD patients tended to have higher D/D genotype frequencies than controls. Interestingly, D allele carriage (D/D+I/D genotypes) was significantly increased in patients compared with controls, and there was a strong association between D allele carriage and elevated APR. Conversely, there was no difference in *ACE* polymorphisms in patients with normal APR levels compared with controls. Moreover, there was no difference in the distribution of *ACE* I/D polymorphism frequencies in RHD patients with high and normal baseline APR levels. D allele carriage was also associated with mitral valve involvement but not CVLs in patients with high APR levels.

The association between the *ACE* I/D polymorphism and RHD has previously been reported in several populations [22], but the results were inconsistent. With respect to Arab populations, a study from Egypt reported an association between the DD genotype and RHD [25], and another study from Saudi Arabia reported a weak association with RHD [24]. Two studies from Turkey and Taiwan reported an association with the I/I genotype. Our current results therefore confirm that the association between the *ACE* I/D polymorphism and disease stratification is stronger than the association with the disease as a whole. Therefore, *ACE* may play an important role as a modifier rather than a causative gene in RHD.

**Figure 2:**
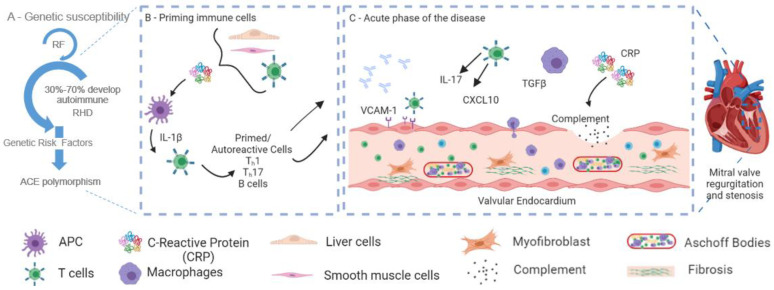
A schematic showing the possible role of C-reactive protein (CRP) in the pathogenicity of rheumatic heart disease (RHD) after group A streptococcal (GAS) infection. (**A**) Rheumatic fever (RF) and RHD are thought to be initiated by infection with rheumatogenic strains of GAS in susceptible hosts [5]. (**B**) An increased level of ACE leads to high angiotensin II, which induces high CRP [32,33]. In turn, CRP induces T cells to express high levels of interleukin-1β (IL-1 β) [34,38]. IL-1 β plays an important role in activating inflammasomes which has been shown to play an important role in RHD [35]. (**C**) Another pathogenic effect of high CRP is its ability to induce heart tissue damage by activating complement [36], which is known to play a role in RHD pathogenicity as well. Figure reprinted/adapted with permission from Ref. Abdallah et al., 2021 [5] and Carlus et al., 2020 [39]. The figure was originally designed using Biorender.com (please see reference [5]).

The relationship between *ACE* polymorphisms and APR levels has been investigated in other diseases. For instance, in chronic obstructive pulmonary disease, the *ACE* I/D polymorphism was associated with high CRP levels [30]. However, whether this represents a direct or an indirect association between D allele carriage and increased inflammatory responses remains unclear. Cross-talk between the RAS system and CRP is well described [31]; ACE is responsible for the conversion of Ang I to the vasoconstrictor Ang II, and in turn, Ang II can induce CRP in vascular smooth muscle cells to stimulate CRP production in aortic endothelial cells [32,33]. In turn, CRP induces T cells to express high levels of interleukin-1β (IL-1 β) [34]. IL-1 β plays an important role in activating inflammasomes, which has been shown to play an important role in RHD [35]. Another pathogenic effect of high CRP is its ability to induce heart tissue damage by activating complement [36], which is known to play a role in RHD pathogenicity as well. Therefore, our results may indicate a direct effect of the D allele on CRP levels, although other studies have failed to detect this association, such as in coronary artery disease, where no association was detected between RAS polymorphisms (including *ACE* I/D) and CRP levels [37]. Figure 2 presents a schematic that shows the possible role of CRP in the pathogenicity of RHD.

The effect of *ACE* polymorphisms on other inflammatory markers has been widely reported. For example, in myocardial infarction patients, the *ACE* ID/DD genotype was independently associated with high IL-6 and kallikrein, a protease that plays a significant role in initiating and maintaining systemic inflammatory responses [38]. In another study, the *ACE* D/D allele was associated with low nitric oxide metabolites (nitrite and nitrate) in healthy men with high blood pressure [39]. Interestingly, high blood pressure was also associated with the *ACE* D/D genotype in obese youths [40]. In complicated cases with COVID-19, D-dimer levels were significantly higher in patients with D/D and I/D genotypes, indicating a potential biomarker of poor disease outcome [41]. In addition, COVID-19 severity was associated with the *ACE* D/D genotype and with high CRP levels [42]. Moreover, the I/D polymorphism was reported to show sexual dimorphism in young adults, with the DD genotype associated with higher maximal fat oxidation values than the ID/II genotype in men, and the ID/II genotype having higher maximal fat oxidation values in women [43]. In ischemic stroke patients, the *ACE* D allele was associated with high total cholesterol and low-density lipoprotein levels, indicating a possible biomarker for early prevention [44]. In cardiovascular disease patients, the *ACE* I/I genotype and I allele were associated with increased circulating glucose levels and hence a 2-fold increased risk of diabetes [45]. These studies indicate that the *ACE* I/D polymorphism has a biological influence.

Baseline ESR and CRP levels have been reported to be influenced by different genetic factors in different diseases. In familial Mediterranean fever, APR levels were significantly higher in children with homozygous and heterozygous M694V mutations compared with those with the wildtype genotype; therefore, monitoring APR levels in patients harboring the mutation may help to prevent disease complications [46]. In rheumatoid arthritis, an intronic variant of *ANKRD55* was associated with the disease, and there was a significant correlation between CRP levels and the variant [47]. Elevated ESR and CRP levels are an important marker for the acute stage of Kawasaki disease (KD) [48], and a polymorphism in *ITPR3*, whose protein product mediates the release of intracellular calcium, was associated with increased serum CRP levels in KD patients. Interestingly, the polymorphism was not associated with the risk of KD, but rather the disease complications [49]. In a GWAS, two polymorphisms were associated with CRP levels in KD patients in both the test and validation cohorts [50]. The first variant (rs4786091) was an intronic polymorphism found in an RNA-binding protein gene, RNA binding Fox-1 homolog 1 (*RBFOX1*), which plays a role in tissue-specific alternative splicing [51]. *RBFOX1* has been associated with the risk of allergy [52], neurodevelopmental disorders [53], and aggressive behavior [54]. The second variant (rs120687530) was a common intergenic polymorphism in the promoter region of *CRP*. However, more recent studies have found that different genotypes and haplotypes in the promoter region of *CRP* are associated with its protein expression [55]. In addition, in a familial study, the heritability of serum *CRP* levels was found to be quartile-specific, namely age and sex, which may explain the different associations between CRP levels and different genes in different diseases [56].

Baseline ESR levels are also influenced by different genetic factors in different diseases. A promoter polymorphism in IL-10 (rs1800871) was associated with increased CRP and ESR levels in patients with spinal tuberculosis, a complication of *Mycobacterium tuberculosis* infection [57]. Moreover, an angiopoietin 2 (*ANGPT2*) rs3020221 polymorphism was found to influence both CRP and ESR levels in rheumatoid arthritis patients. Interestingly, rs3020221 was associated with elevated CRP and ESR levels and Disease Activity Score-28 (DAS-28) ESR scores [58,59]. Similarly, the rs562929801 polymorphism in miRNA-5189 was significantly associated with DAS-28-ESR scores in rheumatoid arthritis patients treated with methotrexate [60]. Another polymorphism in *SPRED2* (rs934734) was associated with an elevated ESR in rheumatoid arthritis patients [61]. Vitamin D receptor (VDR) polymorphisms also impact ESR levels, and in systemic sclerosis patients, BglI and ApaI polymorphisms were associated with an increased ESR [62], while the BsmI polymorphism was associated with an increased ESR in rheumatoid arthritis patients [63].

Our study has a few limitations. One limitation is the limited sample size. In addition, we analyzed APR as a whole rather than analyzing CRP and ESR individually, as these data were not available in the patient records. Finally, we did not investigate the exact mechanisms underlying this association in RHD.

## 5. Conclusions

In conclusion, the role of the ACE I/D genotype in different phenotypes is important to consider in the context of complex, multi-factorial diseases. Our results revealed a strong association between the ACE I/D polymorphism and increased baseline APR levels in children with RHD. These results confirm the importance of ACE I/D polymorphisms in RHD disease stratification, although they carry less influence in disease predisposition. However, whether this association is direct or indirect remains unclear. Given the scarcity of data in this area, further studies in larger cohorts and in different populations are required to confirm this association and to explore the mechanism underlying this effect.

## Figures and Tables

**Table 1:** Demographic characteristics of the patient and control groups (patients *n* = 123, controls *n* = 198).

Parameter			Value
Age (mean ± SD years):			
	Controls		20.5 (4.2)
	Patients		19 (5)
Gender (male/female %):			
	Controls		49.6/50.4
	Patients		56/44
Clinical presentation (*n* (%)):			
	Valvular lesion:		
		Mitral valve lesion (MVL)	67 (54)
		Combined valve lesion (CVL)	57 (46)
	Carditis		79 (64)
	Arthritis		71 (57)
	Chorea		6 (8)
	Skin rash		3 (4)
	Subcutaneous nodules		2 (2.7)
	Recurrence		NA
Laboratory findings (*n* (%)):			
	Elevated acute-phase reactants (CRP/ESR)		75 (75.7)
	Prolonged PR interval		38 (50.7)

Abbreviations: CRP, C-reactive protein; ESR, erythrocyte sedimentation rate; *n*, number; SD, standard deviation; NA, not available; PR, retrograde P waves. There was no significant difference in the sex distribution between the control and patient groups (*p* = 0.24).

**Table 2:** Analysis of ACE I/D polymorphism genotypes and allele frequencies in RHD patients and control group.

Genotype	Control (*n* = 198)		Patients (*n* = 123)				
	Count	Frequency	Count	Frequency	*X^2^*	*p*-Value	OR (95%CI)
DD	88	0.44	60	0.49	5.2	0.07	
ID	85	0.43	57	0.46			
II	25	0.13	6	0.05			
D	261	0.66	177	0.72	3.8	0.06	
I	135	0.34	63	0.28			
DD+ID	173	0.87	117	0.95	4.4	**0.04**	2.9 (1.1–7.6)
II	25	0.13	6	0.05			
II+ID	110	0.56	63	0.51		0.5	
DD	88	0.44	60	0.49			

Significant values are shown in bold. RHD, rheumatic heart disease; *n*: number of cases and controls; OR, odd ratio; 95%CI, 95% confidence interval; D, deletion allele; I, insertion allele.

**Table 3:** Analysis of the genotypes, allele frequencies, and allele carriage distributions in RHD patients with high APR levels, those with normal APR levels, and controls.

Genotype	Control (*n* = 198)		RHD with High APR (*n* = 75)		RHD with Normal APR (*n* = 48)				
	Count	Frequency	Count	Frequency	Count	Frequency	*p*-Value ^1^	*p*-Value ^2^	*p*-Value ^3^
D/D	88	0.44	40	0.53	20	0.43	**0.04**	0.2	0.5
I/D	85	0.43	33	0.44	24	0.52			
I/I	25	0.13	2	0.03	2	0.04			
D	261	0.66	113	0.75	64	0.70	**0.04**	0.5	0.4
I	135	0.34	37	0.25	28	0.30			
DD+ID	173	0.87	73	0.97	44	0.96	**0.02**	0.2	1
II	25	0.13	2	0.03	2	0.04			
II+ID	110	0.56	35	0.47	26	0.57	0.2	1	0.4
DD	88	0.44	40	0.53	20	0.43			

Significant values are shown in bold. Abbreviations: *n*, number; RHD, rheumatic heart disease; APR, acute-phase reactant; D, deletion allele; I, insertion allele. ^1^ Comparison between high APR and control. ^2^ Comparison between normal APR and control. ^3^ Comparison between high and normal APR subgroups.

**Table 4:** Association between *ACE* variants and subgroups of patients with elevated APR (MVL, CVL).

Genotype	Control (*n* = 198)		Patients (*n* = 75)						
			MVL (*n* = 33)		CVL (*n* = 42)		*p*-Value ^1^	*p*-Value ^2^	*p*-Value ^3^
	Count	Frequency	Count	Frequency	Count	Frequency			
D/D	88	0.44	18	0.55	22	0.52	0.06 *	0.3 *	0.4 *
I/D	85	0.43	15	0.45	18	0.43			
I/I	25	0.13	0	0	2	0.05			
D	261	0.66	51	0.77	62	0.74	0.09	0.2	0.8
I	135	0.34	15	0.23	22	0.26			
D/D+I/D	173	0.87	33	1.0	40	0.95	0.02 *	0.1 *	0.3 *
I/I	25	0.13	0	0	2	0.05			
II+ID	110	0.56	15	0.45	20	0.48	0.2	0.2	0.5
DD	88	0.44	18	0.55	22	0.52			

Significant values are shown in bold. Abbreviations: MVL, mitral valve lesion; CVL, combined valve lesion; *n*, number; APR, acute-phase reactant; D, deletion allele; I, insertion allele. All analyses were conducted using the chi-squared test, except for tests with (*), where Fisher’s exact test was used. ^1^ Comparison between MVL subgroup and controls. ^2^ Comparison between CVL subgroup and controls. ^3^ Comparison between MVL and CVL subgroups.

## Data Availability

The data presented in this study are available on request from the corresponding author.
